# Low-Temperature-Mediated Promoter Methylation Relates to the Expression of *TaPOR2D*, Affecting the Level of Chlorophyll Accumulation in Albino Wheat (*Triticum aestivum* L.)

**DOI:** 10.3390/ijms241914697

**Published:** 2023-09-28

**Authors:** Jingjing Du, Junwei Wang, Sicong Shan, Tian Mi, Yulong Song, Yu Xia, Shoucai Ma, Gaisheng Zhang, Lingjian Ma, Na Niu

**Affiliations:** 1 College of Agronomy, Northwest A & F University, Xianyang 712100, China; dou0321@nwafu.edu.cn (J.D.); wjw@nwsuaf.edu.cn (J.W.); ssc0116266@163.com (S.S.); 18734253981@163.com (T.M.); sylbl1986@nwsuaf.edu.cn (Y.S.); xiayu325@nwafu.edu.cn (Y.X.); mashoucai@sohu.com (S.M.); zgs02196@163.com (G.Z.); 2 Key Laboratory of Crop Heterosis of Shaanxi Province, Xianyang 712100, China; 3 Wheat Breeding Engineering Research Center of Ministry of Education, Xianyang 712100, China

**Keywords:** wheat, methylation, chlorophyll, NADPH protochlorophyllide oxidoreductase

## Abstract

Chlorophyll is an indispensable photoreceptor in plant photosynthesis. Its anabolic imbalance is detrimental to individual growth and development. As an essential epigenetic modification, DNA methylation can induce phenotypic variations, such as leaf color transformation, by regulating gene expression. Albino line XN1376B is a natural mutation of winter wheat cultivar XN1376; however, the regulatory mechanism of its albinism is still unclear. In this study, we found that low temperatures induced albinism in XN1376B. The number of chloroplasts decreased as the phenomenon of bleaching intensified and the fence tissue and sponge tissue slowly dissolved. We identified six distinct *TaPOR* (protochlorophyllide oxidoreductase) genes in the wheat genome, and *TaPOR2D* was deemed to be related to the phenomenon of albinism based on the expression in different color leaves (green leaves, white leaves and returned green leaves) and the analysis of promoters’ cis-acting elements. TaPOR2D was localized to chloroplasts. *TaPOR2D* overexpression (TaPOR2D-OE) enhanced the chlorophyll significantly in Arabidopsis, especially at two weeks; the amount of chlorophyll was 6.46 mg/L higher than in WT. The methylation rate of the *TaPOR2D* promoter in low-temperature albino leaves is as high as 93%, whereas there was no methylation in green leaves. Correspondingly, three DNA methyltransferase genes (*TaMET1*, *TaDRM* and *TaCMT*) were up-regulated in white leaves. Our study clarified that the expression of *TaPOR2D* is associated with its promoter methylation at a low temperature; it affects the level of chlorophyll accumulation, which probably causes the abnormal development of plant chloroplasts in albino wheat XN1376B. The results provide a theoretical basis for in-depth analysis of the regulation of development of plant chloroplasts and color variation in wheat XN1376B leaves.

## 1. Introduction

Photosynthesis is the process by which green plants convert light energy into chemical energy [1,2]. The photosynthetic efficiency is affected by the type and content of pigments in leaves, since leaves are the main organ of photosynthesis. Chlorophyll is one of the main pigments involved in photosynthesis, and it plays a crucial role in plant growth and development [3].

Chlorophyll synthesis is an extremely complex biological process, which includes three main steps: chlorophyll biosynthesis, chlorophyll cycle and chlorophyll degradation [4,5,6]. The biosynthesis of chlorophyll molecules starts from L-glutamyl-tRNA and produces chlorophyll a and chlorophyll b with the participation of different enzymes. The whole biosynthesis process includes 15 steps and requires 15 enzymes. At present, 27 genes encoding these enzymes have been identified and cloned from the model plant Arabidopsis thaliana, completing the cloning of all genes involved in the chlorophyll biosynthesis process [7].

Protochlorophyllide oxidoreductase (NADPH; POR, EC 1.3.1.33) is a major component of the yellowing plastid membrane protein [8]. Precursors in the early stages of plants form yellowing plastids under dark conditions and develop into chloroplasts after illumination. In this process, POR catalyzes the photoreduction of protochlorophyllide (Pchlide) a to chlorophyllide a, and it is present at high levels as a ternary complex with Pchlide and NADPH, forming prolamellar bodies (PLBs) in etioplasts of dark-grown seedlings [9]. Thus, POR plays the key role in the development of plant light morphology and participates in the 13th step reaction of chlorophyll synthesis [10,11,12].

The number of POR isoforms differs among angiosperms. There are three PORs in Arabidopsis; two PORs in rice, barley and tobacco; and one in cucumber [13]. In addition, the expression pattern and function of PORs are quite different in the different species, even in the same species. For example, HvPORA and HvPORB show similar enzymatic activities in vitro, but the expression patterns of *HvPORA* and *HvPORB* in etiolated barley seedlings are different from each other [14]. *AtPORB* and *AtPORC* genes are strongly expressed early in seedling development. Overexpression of PORB can significantly alter chlorophyll content in transgenic plants and have an important regulatory effect on the development of plant chloroplasts [15,16,17,18]. However, so far, there is little information on the isoforms and function of POR in wheat.

Albino is a type of leaf color variation, and it is caused by the loss of chlorophyll or chloroplast formation obstruction. If chlorophyll is lacking in the albino plants, photosynthesis is abnormal. The young seedlings grow through the endosperm or cotyledon. When the nutrients are exhausted, the plants die. At present, leaf albininism has been observed in a variety of plants, such as *Oryza sativa*, *Arabidopsis thaliana*, *Triticum aestivum*, *Nicotiana tabacum*, *Zea mays* and *Hordeum vulgare* [19,20,21,22,23,24,25]. Leaf color mutants are often used to explore the mechanism of photosynthesis, chlorophyll biosynthesis, chloroplast structure and function, and genetic development regulation. Studying the mechanism of leaf albininism has theoretical and practical importance in plants. As for the causes of leaf bleaching, multiple regulatory pathways and metabolic processes, such as hormone imbalance, mutations in chloroplast genes, and chlorophyll biosynthesis genes mutations have been examined. However, the mechanism of bleaching is not well understood.

DNA methylation is important in epigenetic regulation during plant growth and development [26,27]. Current research on plant DNA methylation focuses on the methylation of nuclear genomic DNA, including cytosine methylation (5 mC) and adenine methylation (6 mA) [28,29,30]. The level and state of cytosine methylation of nuclear genomic DNA in plants changes under external stress conditions such as salinity, heavy metals, drought, low temperature, pathogen infestation, regulating gene expression and plant stress response [31]. 

Studies have shown that Chlamydomonas 6 mA is a marker of gene transcription activity and expression, which is very different from the location and related functions of 5 mC; 6 mA promotes the initiation of gene transcription, and 5 mC inhibits gene expression [32,33]. The degree of methylation of cytosine DNA is affected by low temperature stress, but the results vary in different types of plants. Studies of Arabidopsis [34], jade rice [35], wheat [36] and other plants have shown that low temperature causes a decrease in the degree of nuclear genomic methylation, resulting in high expression of low-temperature response genes and anti-stress-related genes. In rice, the methylation level of the inflorescence of the hardy cultivar LTH was down-regulated and the level of root methylation was up-regulated at a low temperature. The methylation level of the inflorescence of low-temperature-sensitive cultivar IR64 was also down-regulated, and the methylation level of the leaves was significantly increased after undergoing low-temperature stress, indicating that methylation was tissue-specific [36]. Due to the hormone response under adverse conditions causing histone modification, the leaf colors of wheat were albino and green leaf on different tillers of the same plant [37]. During the leaf color transformation process of *Photinia rubiana*, the methylation level of its genome changed.

In this study, the POR family was identified in wheat. Albino line XN1376B, a natural mutation of winter wheat cultivar XN1376, was used as the test material. We identified a key enzyme gene *TaPOR2D* in the wheat chlorophyll synthesis pathway that was related to the albino phenotype based on its expression in different colored leaves (green leaves, white leaves and returned green leaves) and analysis of promoter cis-acting elements. We investigated the organizational structure of leaves and level of chlorophyll in response to low temperature, as well as the function of *TaPOR2D* in chlorophyll accumulation by overexpressing it in transgenic plants. Did promoter methylation of *TaPOR2D* contribute to albinism at low temperatures? How did bleaching happen? To solve these two problems, we determined the leaf microstructure, expression level of *TaPOR2D* and the methylation level in the promoter region of *TaPOR2D* for white leaves and green leaves. We also measured the expression level of three methyltransferases genes, *TaMET1*, *TaCMT*, *TaDRM* in albino line XN1376B, at low temperatures. We verified the level of chlorophyll in transgenic Arabidopsis. The results showed that the expression of *TaPOR2D* was associated with its promoter methylation at a low temperature; it affected the level of chlorophyll accumulation, which probably caused the abnormal development of plant chloroplasts in albino wheat XN1376B. The results provide a theoretical basis for in-depth analysis of the regulation of the development of plant chloroplasts and color variation in XN1376B wheat leaves.

## 2. Results

### 2.1. Phenotypic Characteristics of XN1376B

**Figure 1:**
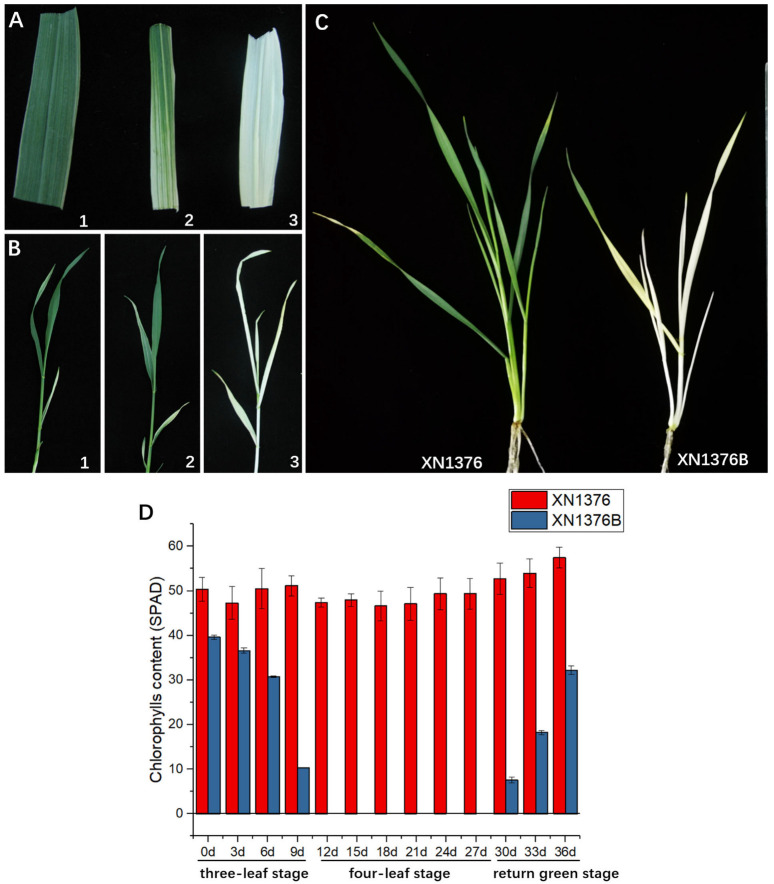
Phenotype and chlorophyll of XN1376 and XN1376B. (**A**,**B**) XN1376B change characteristics. 1: two-leaf stage; 2: three-leaf stage; 3: four-leaf stage. (**C**) Overwintering stage of XN1376 and XN1376B. (**D**) Chlorophyll of XN1376 and XN1376B.

In the field, when the temperature is 5–10 °C, white stripes began to appear at the three-leaf stage of XN1376B (Table 1, Figure 1A,B). The white leaves became more pronounced at the seventh day after the onset of bleaching, and the plant became all white at the four-leaf stage when grown at about 5 °C (Figure 1A,B). At the regreening stage of wheat, the leaves of XN1376B began to turn green with the increase in atmospheric temperature (Table 1). However, XN1376 always stayed green (Table 1, Figure 1C).

Atmospheric temperatures were simulated in an incubator, and three-leaf stage seedlings of XN1376B and XN1376 were treated at 5 °C for four weeks, followed by 12 °C for one week. XN1376B wheat gradually turned white in the first week under 5 °C conditions, and the seedlings were completely bleached after two weeks; when the temperature rose to 12 °C, XN1376B wheat began to re-green.

Meanwhile, the level of leaf chlorophyll was measured using a portable chlorophyll meter during the growth and development of the wheat. The results showed that there was a significant difference in chlorophyll between XN1376 and XN1376B throughout the bleaching stage (Figure 1D). The chlorophyll remained stable through all stages in XN1376. However, in XN1376B, it decreased with increased bleaching and bounced back with the return of greening. It is worth noting that the XN1376B was completely albino from the 12th to 27th day after the onset of bleaching.

The above results indicate that chlorophyll synthesis is inhibited at low temperature in XN1376B.

### 2.2. Histological Analysis of XN1376B with Different Leaf Colors

**Figure 2:**
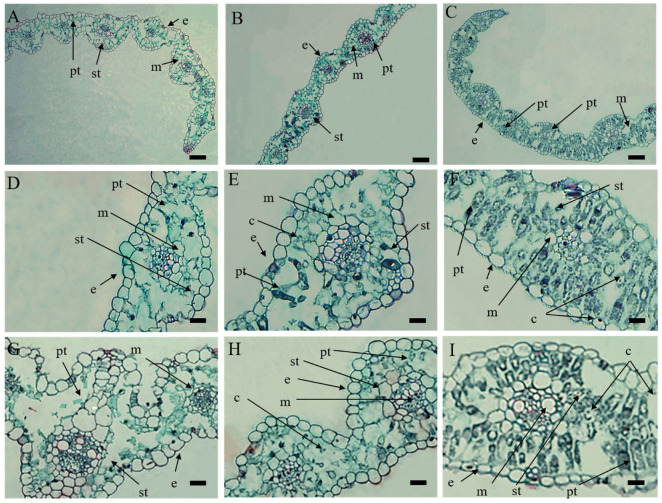
Microstructure of mesophyll cells in XN1376B. (**A**,**D**,**G**) White leaves of XN1376B. (**B**,**E**,**H**) Return-green leaves of XN1376B. (**C**,**F**,**I**) Green leaves of XN1376; (e) epidermis; (m) mesophyll; (c) chloroplast; (pt) palisade tissue; (st) spongy tissue. Scale bar: 200 µm in (**A**–**C**), 100 µm in (**D**–**I**).

In green leaves of XN1376, the tissue structure was complete. Mesophyll cells were neatly arranged. We observed normal palisade tissue, spongy tissue and a large number of chloroplasts in the cells (Figure 2C,F,I). The similar tissue structures were observed in return-green leaves of XN1376B (Figure 2B,E,H). However, in white leaves, although the epidermal cells and mesophyll cells of leaves were not significantly different from those of green leaves, palisade tissues and spongy tissues of white leaves became shapeless and dissolved, and chloroplasts almost disappeared. (Figure 2A,D,G). The structure of vascular bundle sheath cells was similar in both green and white leaves.

### 2.3. Identification of NADPH: Protochlorophyllide Oxidoreductase (POR) Gene in Wheat

All publicly known POR protein sequences of Arabidopsis, rice, maize, soybean and common bean were used as queries in BLASTP searches against the wheat genome database included in EnsemblPlants (http://plants.ensembl.org, accessed on 10 June 2021) and the NCBI database (https://www.ncbi.nlm.nih.gov/, accessed on 10 June 2021). Six wheat POR genes (*TaPOR1A*, *TaPOR1B*, *TaPOR1D*, *TaPOR2A*, *TaPOR2B* and *TaPOR2D*) were identified in wheat by a genome-wide analysis. Six *TaPOR*s were collapsed into two distinct wheat genes and were renamed based on their sequence characteristics and their order of distribution on the wheat chromosome in this study (Table S1). Six *TaPOR*s contained redundant homologous copies from sub-genomes A, B and D (called triplets). *TaPOR*s encoded 388–417 amino acids, with a molecular weight of 41.15–44.72 kDa and 9.15–9.42 isoelectric points. Subcellular localization predictions revealed that proteins functioned in chloroplasts (Table S1).

### 2.4. Gene Structure and Evolutionary Relationship of TaPORs

**Figure 3:**
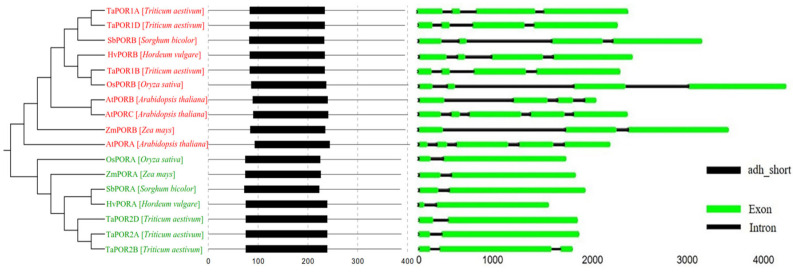
Phylogenetic, conserved motifs and gene structure of POR families from wheat, sorghum, barley, rice, maize and Arabidopsis. Green font: PORA; Red font: PORB.

A total of 17 POR protein sequences from *Arabidopsis thaliana* L., *Sorghum bicolor* L., *Hordeum vulgare* L., *Oryza sativa* L., *Zea mays* L. and *Triticum aestivum* L. were used to construct a phylogenetic tree (Figure 3). The POR proteins were clustered into two groups (PORA, PORB). The PORA comprised one OsPORA, one HvPORA, one SbPORA, one ZmPORA and three TaPORA proteins (TaPOR2A, TaPOR2B, TaPOR2D), which may have similar functions. The PORB comprised two AtPORB, one OsPORB, one HvPORB, one SbPORB, one ZmPORB, one AtPORA and three TaPORB proteins (TaPOR1A, TaPOR1B, TaPOR1D).

The POR proteins of different species contained a primary domain adh_short, which was similar in location and size. For the POR gene structure, the same group of structures had a similar number, size of exons and sequence. Different groups had slightly different gene structures. TaPORs protein sequence similarity was 82.06%; the similarity of the three homologous copy proteins in TaPORAs was 97.28%, and the similarity of TaPORBs was up to 99.05%. Within the adh_short domain, TaPORAs had a 99.60% similarity, whereas TaPORBs were exactly the same. All TaPORs contain NADPH-binding motif (YKDSK) and active site motif (TGASSGLG) (Figure S1).

### 2.5. Expression Pattern of TaPOR Genes in Wheat XN1376B

**Figure 4:**
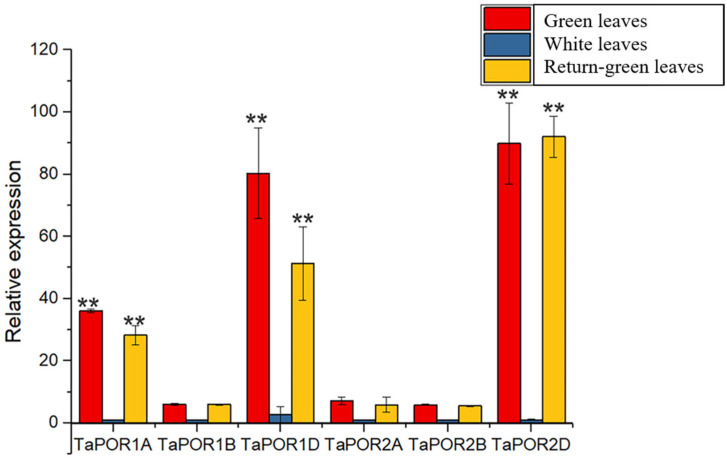
Expression profiles of 6 *TaPOR* genes of XN1376B in green leaves, white leaves and return-green leaves. Data represent means ± SD (*n* = 3). Each column represents the mean ± standard error based on three biological repeats. Significant differences were determined by Student’s test (** *p* ≤ 0.01).

In order to investigate whether the *TaPOR*s are involved in the regulation of leaf colors, three kinds of leaves from XN1376 and XN1376B were used to analyze the expression pattern of the *TaPOR*s via qRT-PCR (Figure 4). *TaPOR* genes had different expression profiles among different colored leaves. All the *TaPOR*s had higher expression levels in the green leaves and recovering green leaves. *TaPOR1A*, *TaPOR1D* and *TaPOR2D* showed a more significant expression difference. *TaPOR2D* exhibited the largest transcript-level differences with high expression levels in green leaves and return-green leaves and almost no expression in white leaves.

### 2.6. Promoter Analysis of TaPORs

**Figure 5:**
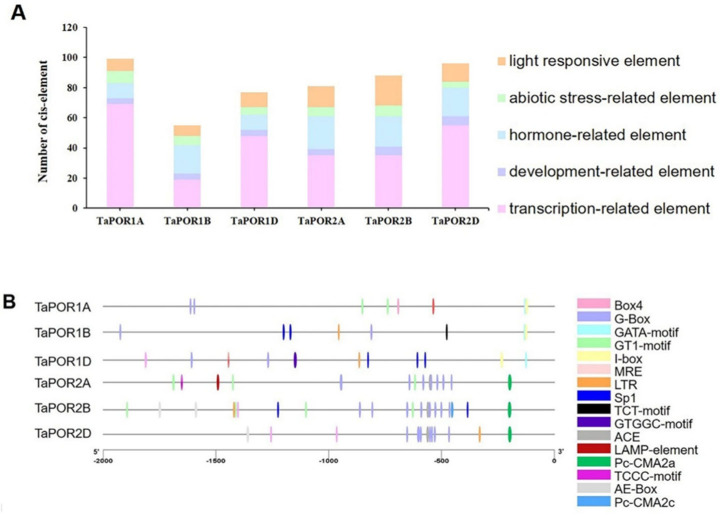
Main cis-elements in TaPOR gene promoters. (**A**) Light-responsive, abiotic stress-related, hormone-related, development-related and transcription-related elements were identified in the TaPOR gene promoter regions. (**B**) Location of 16 types of light-response elements and cryogenic elements in the TaPORs promoter region.

Cis-acting elements in gene promoters are crucial in transcription factor binding for the initiation of transcription. We cloned the promoters of six *TaPOR* genes from XN1376 and XN1376B (Figure S2). Here, 2000bp upstream promoter regions from all *TaPOR*s were used to identify putative cis-acting elements. We found phylogenetically similar genes shared identical cis-elements. Five types of cis-acting elements including transcription-related, light-responsive, development-related, hormone-related and abiotic stress-related elements were identified (Figure 5A). All of the *TaPOR* promoters contained a certain number of transcription-related elements, such as TATA-box and CAAT-box. Light-responsive elements were also included, such as the BOX4 (ATTAAT), G-BOX (CACGTG), GATA-motif (GATAGGG), GT1-motif (GGTTAA), I-box (GATAAGGTG), MRE (AACCTAA), Sp1 (GGGCGG), TCT-motif (TCTTAC), GTGGC-motif (GATTCTGTGGC), ACE (GACACGTATG), LAMP-element (CTTTATCA), Pc-CMA2a (CAGCCAATCACAG), TCCC-motif (TCTCCCT), AE-Box (AGAAACAA) and Pc-CMA2c (GCCCACGCA). Abiotic stress-related elements included low-temperature element (LTR; CCGAAA), TC-rich element (GTTTTCTTAC), MBS (CAACTG) and GC-motif (CCCCCG). Hormone-related elements included MeJA-responsive element (TGACG-motif, CGTCA-motif), ABRE (ACGTG, CGTACGTGCA), TGA-element (AACGAC) and GARE-motif (TCTGTTG).

In particular, the number, type and position of light-responsive elements and LTR were different among the six *TaPOR*s (Figure 5B). LTR mainly existed in the promoters of *TaPOR2B*, *TaPOR2D* and *TaPOR1B*. There was no LTR in the promoters of *TaPOR2A* and *TaPOR1A*. There were 8, 7, 10, 14, 20 and 12 light-responsive elements in *TaPOR1A*, *TaPOR1B*, *TaPOR1D*, *TaPOR2A*, *TaPOR2B* and *TaPOR2D*, respectively. The number of light-responsive elements in *TaPORA* was significantly greater than in *TaPORB*. These results further illustrate that *TaPOR* transcript levels might be regulated by light or temperature signals, especially for TaPORAs (*TaPOR2A*, *TaPOR2B*, *TaPOR2D*). Combined with the expression analysis, *TaPOR2A*, *TaPOR2B* showed the lower expression level, whereas *TaPOR2D* showed higher expression in white leaves. Therefore, we selected *TaPOR2D* for further investigation.

### 2.7. Subcellular Localization of TaPOR2D Proteins

**Figure 6:**
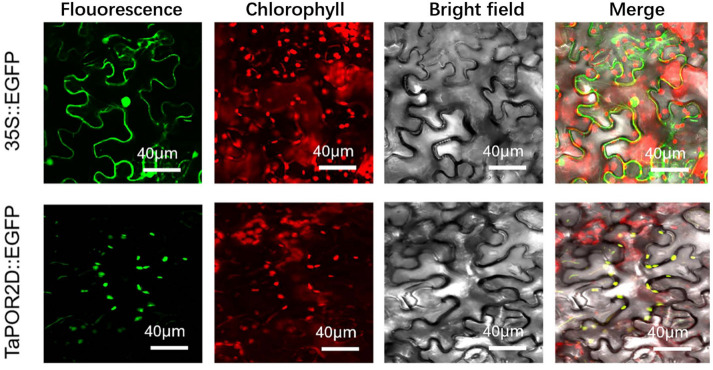
Subcellular localization of TaPOR2D fused with EGFP in the epidermal cells of N. benthamiana. TaPOR2D was cloned from XN1376 and used to construct CaMV35S::TaPORs–EGFP vectors in which EGFP was fused at the C-terminus. Bar = 40 μm.

TaPOR2D was predicted to be localized in the chloroplast. The fluorescent protein-tagging method was used to confirm TaPOR2Ds were chloroplast-localized proteins. The results showed that enhanced green fluorescent protein (EGFP) alone presented a dispersed cell distribution. EGFP-tagged TaPOR2D was located in the chloroplast (Figure 6), consistent with our predictions.

### 2.8. Effect of TaPOR2D Overexpression on the Level of Chlorophyll in Arabidopsis

**Figure 7:**
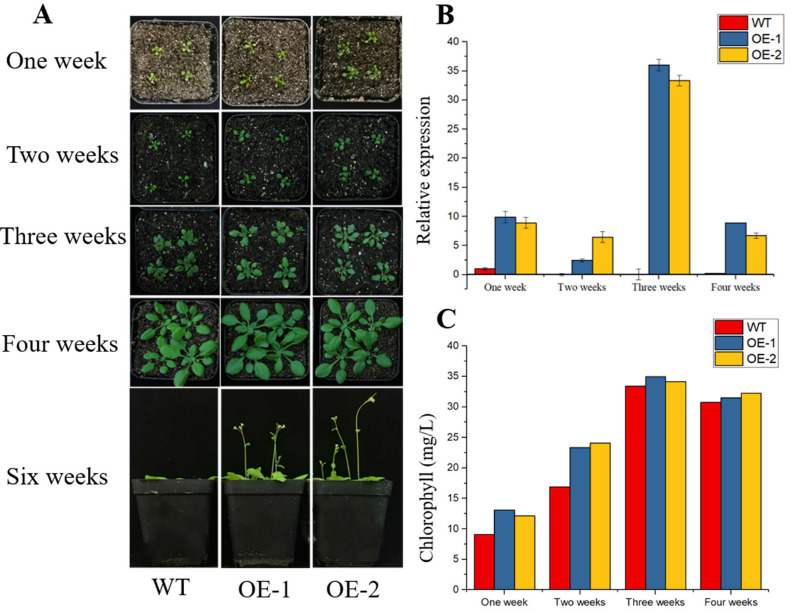
Phenotypic characterization of transgenic and wild-type (WT) Arabidopsis plants. (**A**) Representative images show WT and transgenic lines, after one week of growth, two weeks of growth, three weeks of growth, four weeks of growth and six weeks of growth. (**B**) qRT-PCR identification of transgenic *TaPOR2D* overexpression Arabidopsis plants. *AtActin* was used as an internal control. Data represent means ± SD (*n* = 3). Each column represents the mean ± standard error based on three biological repeats. (**C**) Total chlorophyll in WT, TaPOR2D-overexpressing (OE-1 and OE-2).

*TaPOR2D* was overexpressed in Arabidopsis to confirm its function in chlorophyll biosynthesis. Two homozygous T3 transgenic lines, OE1 and OE2, were used for analysis. Expression levels of *TaPOR2D* were investigated via qRT-PCR. The results showed that *TaPOR*2D was highly expressed in the two transgenic lines, but not expressed in the wild type (WT) (Figure 7B), confirming that *TaPOR2D* was successfully transformed into Arabidopsis.

There was a distinct difference in plant size and leaf color between the wild type (WT) and transgenic lines after one week. Under normal growth conditions, there were larger leaves in the overexpressing lines. Almost all *TaPOR* transgenic plants had greener leaves in the second week compared with WT plants. For three-week-old plants, the color of the transgenic lines’ leaves was still greener than those of WT plants (Figure 7A). After four weeks, there was no major difference in plant growth, except for early flowering time in the overexpressing lines. We further evaluated the chlorophyll level in the leaves of WT and TaPOR-OE (Figure 7C). We found maximum levels of chlorophyll accumulated in the third week in both WT and transgenic lines. However, compared with WT plants, the two transgenic lines showed higher chlorophyll levels at each time point.

### 2.9. Promoter Methylation Analysis of TaPOR2D

The distribution of CpG islands in the promoter regions of *TaPOR2D* was analyzed using MethPrimer online software. We predicted that there was one CpG island of 650 bp in the downstream of the promoter (Figure S3). This putative CpG island was preceded by some functional elements such as a low-temperature-responsive element, a light-responsive element and so on (Figure 5B).

**Figure 8:**
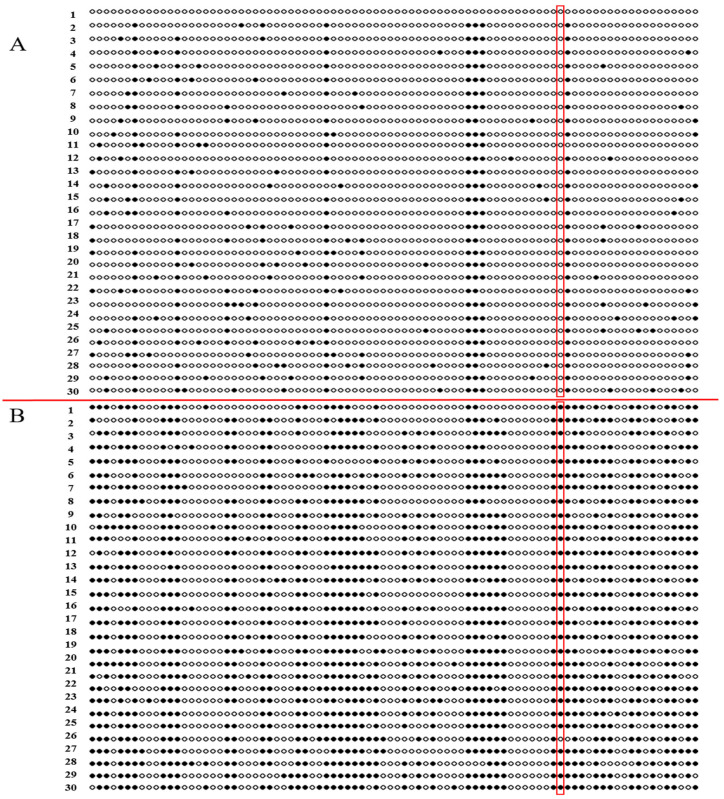
Methylation profiles in the promoter regions of *TaPOR2D*. (**A**) Green leaves. (**B**) White leaves. 1–30: 30 clones of each amplified fragment. White circle: no-methylation loci, black circle: methylation loci, red frame: the differences of methylation at LTR element between green and white leaves.

The methylation level in the predicted region of *TaPOR2D* was determined for white leaves and green leaves via sodium bisulfite treatment and sequencing. In XN1376B, the overall methylation rate of the *TaPOR2D* promoter CpG site in the white leaves was 49%, and in the green leaves this was 12.7%; there were significant differences (*p* < 0.01) between the green leaves and white leaves (Figure 8). The Pearson coefficient between the expression level of *TaPOR2D* and methylation rate was −1, indicating that a significant negative correlation existed between promoter methylation and the gene expression of *TaPOR2D* (Table S2). The analysis of the methylation rate of the LTR element showed that methylation occurred in the LTR (CCGAAA) element in the white leaves but did not occur in the green leaves.

### 2.10. Expression of Methyltransferase Genes at XN1376B

**Figure 9:**
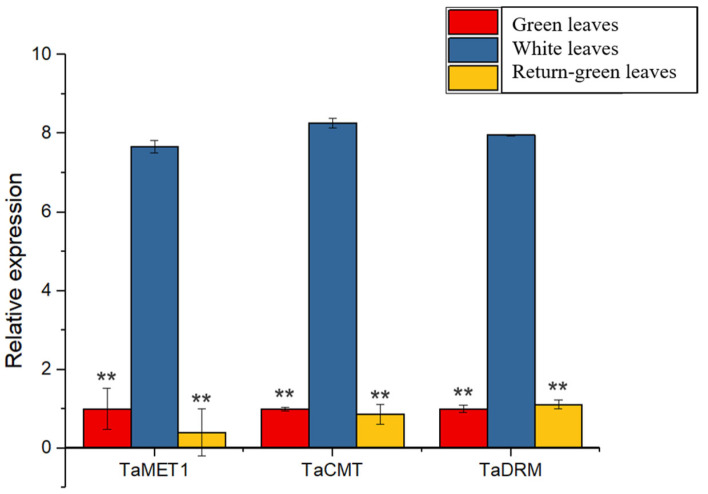
Expression profiles of 3 methyltransferase genes in XN1376B. Quantitative real-time polymerase chain reaction (RT-qPCR) was used to detect the expression levels of the *TaMET1*, *TaCMT*, *TaDRM*. *TaActin* was used as an internal control. Each value represents the mean of three biological replicates ± SE. Significant differences were determined by Student’s test (** *p* ≤ 0.01).

To further examine why DNA methylation could regulate the expression of *TaPOR2D*, the expression of three methyltransferase genes (*TaMET1*, *TaCMT*, *TaDRM*) was determined for white leaves and green leaves. As shown in Figure 9, the average expression of *TaMET1*, *TaCMT* and *TaDRM* in white leaves of XN1376B was higher than that in green leaves.

## 3. Discussion

Albinism is common in plants. Changes in many environmental factors, such as drought and low temperatures, may cause bleaching. Low temperatures inhibit the expression of coding genes to a certain extent, making the albino phenotype more obvious [22]. In addition, deletion mutations in genes such as the ribosome rRNA gene and the chloroplast ribosome protein gene reduce the translation efficiency of chloroplasts by influencing the assembly of ribosomes [23]; mutations in genes associated with cytoplasmic rRNA stabilization and protein translation can also lead to the occurrence of low-temperature-induced albinism.

Theoretically, albinos will eventually not survive because of insufficient chlorophyll synthesis and the plants’ inability to carry out photosynthesis [24]. However, studies have found that some albino mutants are able to survive, and these mutants exhibit staged albinism that is sensitive to environmental conditions [25,31,32]. In this study, white stripes began to appear at the three-leaf stage of XN1376B when the average temperature was below 10 °C in the field. As the temperature continued to drop, the plants turned white completely until the overwintering period was over. When the average temperature rose above 10 °C, XN1376B quickly re-greened and the bleaching phenomenon disappeared. Similar results were observed in the incubator, and the leaf chlorophyll of XN1376B decreased significantly about 7–10 days after the onset of bleaching. Thus, the albino form of XN1376B was induced by the low temperature.

The phenomenon of albinism is summed up as a manifestation of chloroplast dysfunction that causes abrupt changes in the leaf color [38]. Rice albininism was caused by chlorophyll and carotenoid deficiency, and plastids and chloroplasts could not be observed in mesophyll cells [39]. We also observed the obvious differences in the organizational morphological structure between white leaves and green leaves. Compared with the green leaves, the palisade and sponge tissue were partially confused in the white leaves, with only a small number of chloroplasts, although the epidermal cells and mesophyll cells of the white leaves were not significantly different from those in the green leaves. The palisade tissue and sponge tissue of the white leaves became unformed and dissolved, and the chloroplasts almost disappeared.

POR has been well demonstrated as a key protein in chlorophyll synthesis. However, there is little information on the quantity and types of wheat PORs. We identified six TaPOR members from the wheat genome. They have an NADPH-binding motif (YKDSK) and an active site motif (TGASSGLG), similar to those found in Arabidopsis, rice and barley. There were differences in the numbers of amino acids, molecular mass and isoelectric points among TaPOR proteins, which may embody different functions during development. Most of the protein sequences were stable and alkaline, and all proteins were predicted to be located in chloroplasts. Phylogenetic analysis showed that TaPOR family members were divided into two categories, of which TaPORs were more closely related to barley and less related to Arabidopsis. The conserved motifs of the TaPORs, the number of introns, the length of the UTR and the structure of the TaPORs in the different groups were highly similar.

To dissect the expression patterns of *TaPOR* genes in leaves with different colors, we performed real-time fluorescence quantitative expression analysis of six *TaPOR* genes in wheat, and found the expression of 1A, 1D and 2D genes was significantly different among green leaves, white leaves and returned-green leaves, which indicated that the three genes may be closely related to the phenomenon of bleaching in XN1376B wheat. The promoters of the *TaPORs* were amplified and the promoter elements were predicted; we noted that the low-temperature-response element was found only on 1B, 2B and 2D.

PORA and PORB are isoenzymes [18], and the use of RNAi to inhibit the expression of *OsPORA* did not affect the normal synthesis of chlorophyll in rice, whereas *OsPORB* mutants showed a yellowed phenotype, showing that *OsPORB* can complement the deletion of *OsPORA* and is a more critical gene for chlorophyll synthesis. Our results are not consistent with that. Promoter analysis showed that *TaPOR* transcript levels might be regulated by light or temperature signals, especially for *TaPORA* (*TaPOR2B, TaPOR2D*). Combined with the expression result, *TaPOR2A*, *TaPOR2B* showed a lower expression level, whereas *TaPOR2D* showed higher expression in white leaves. Thus, the *TaPOR2D* gene was selected for further investigation. As predicted, TaPOR2D was located in the chloroplast and its overexpression in Arabidopsis can increase chlorophyll accumulation, suggesting it promotes the biosynthesis of chlorophyll.

As a common epigenetic modification in the plant genome, DNA methylation regulated gene expression, which has been confirmed in many studies [40,41]. In our study, the methylation level in the promoter region of *TaPOR2D* was determined to examine the link between DNA methylation and gene expression for white leaves and green leaves. We found that methylation occurred at the LTR element of *TaPOR2D* in leaves of XN1376B, but no methylation occurred in green leaves. Correspondingly, three methyltransferase genes (*TaMET1*, *TaCMT*, *TaDRM*) had high expression in white leaves. The expression of *TaPOR2D* was perhaps regulated by the methylation level in the promoter region. The results provide a theoretical basis for in-depth analysis of the regulation of development of plant chloroplasts and color transformation in XN1376B wheat leaves.

## 4. Materials and Methods

### 4.1. Plant Materials and Growth Conditions

Albino line XN1376B is a natural mutation of winter wheat cultivar XN1376. XN1376B and XN1376 were used in this study. The seeds were grown conventionally in the experimental station of Northwest Agriculture and Forestry University, Yangling, China (108°82′ E, 34°15′ N) to obtain leaves (green leaves, white leaves and return-green leaves) on 10 October 2021. Each row was 1 m, and the row spacing was 0.25 m. We planted the seeds in nutrient pots and watered them daily to avoid drought stress. When the seedlings had grown to the three-leaf stage, they were subjected to cold treatment. In the incubator, we treated three-leaf stage XN1376B and XN1376 at 5 °C for four weeks, then at 12 °C for one week. For determination of the expression pattern of *TaPOR* under low-temperature treatments, the leaves were harvested at 0 day, 10 day, 20 day and 30 day during treatment. All samples were immediately frozen in liquid nitrogen and stored at −80 °C for further analysis.

For the histological structure analysis, approximately 0.3 cm of leaf tissue was obtained from green, white and return-green plants, and these samples were immersed in the 70% FAA solution.

The Arabidopsis thaliana ecotype Columbia (Col-0) used in this study was from our laboratory. The seeds were surface-sterilized at 4 °C for three days following the method described previously [6]. The plates were transferred to the incubator for 7 days under a 16 h light/8 h dark photoperiod at 22 °C. Seedlings were grown for three weeks and then subjected to cold treatment (under a 16 h light/8 h dark photoperiod at 8 °C).

### 4.2. Histological Examination of the Cells of Different Colored Leaves

For paraffin sectioning, the samples fixed in 70% FAA were removed and rinsed clean and then were placed into ethanol solutions with different concentrations (70%, 85%, 95%, and 100%) for 30 min to dehydrate. Next, after they had been placed in 100% ethanol for 30 min twice, the samples were placed in a mixture solution (50% ethanol and 50% xylene) for 1 h, and then were put into a pure xylene solution for 1 h twice, followed by immersion in a mixture solution (50% xylene and 50% paraffin) for 12 h at 37 °C overnight. Thereafter, they were placed in an oven at 55–60 °C for paraffin embedding, and the freshly melted paraffin solution was replaced every 4 h. The paraffin embedded leaf segments and melted paraffin were rapidly poured into an embedding box, followed by fixation and embedding in ice water. The samples were cut into 8-μm-thick sections on a rotary 109 rotary microtome (YD-2508A; Jinhua YIDI Medical Appliance Co., Jinhua, China). The sections were stained with safranin and Fast Green and photographed using a digital camera (Eclipse E600; Nikon, Tokyo, Japan) [42].

### 4.3. Extraction and Determination of Chlorophyll

For the measurement of total chlorophyll concentration, pigments were extracted from leaf tissues of wild-type Arabidopsis and *TaPOR* overexpression Arabidopsis with 80% ice-cold acetone. The concentrations of chlorophyll were determined with a UV/VIS spectrophotometer [43]. Then, 100 mg samples of the leaf tissues were cut in the dark room and incubated in 10 mL of 80% acetone in darkness for 12 h at 25 °C. After centrifugation, the supernatant was collected and absorbance was measured at 663 nm and 647 nm using a spectrophotometer. Chlorophyll a and chlorophyll b in the leaf samples were calculated according to Arnon [44].

### 4.4. Identification of POR Subfamily Genes in Wheat and Bioinformatics Analysis

All publicly known POR protein sequences of Arabidopsis, rice, maize, soybean and common bean were used as queries in BLASTP searches against the wheat genome database included in EnsemblPlants (http://plants.ensembl.org, accessed on 10 June 2021) and the NCBI database (https://www.ncbi.nlm.nih.gov/, accessed on 10 June 2021). Three *TaPORA* (*TaPOR1A*, *TaPOR1B* and *TaPOR1D*) and three *TaPORB* (*TaPOR2A*, *TaPOR2B* and *TaPOR2D*) putative cDNA sequences of genes associated with *PORB* and *PORA* were found in wheat.

A phylogenetic tree was constructed using ClustalX and MEGA X. Prediction of GRAVY was performed using the online tool ProtScale (http://www.gravycalculator.de/, accessed on 15 June 2021). Logo diagrams used to define consensus sequences were obtained using multiple sequence alignments for TaPORs by TEXshade. The online tool SMART (http://smart.embl-heidelberg.de/, accessed on 15 June 2021) was used for conserved domains analysis of *TaPORs*. Prediction of physical and chemical parameters was undertaken using the ProtParam tool (http://web.expasy.org/protparam/, accessed on 15 June 2021).

### 4.5. DNA and RNA Extraction, cDNA Synthesis and qRT-PCR Analysis

The total DNA was extracted via the New Genomic DNA Extraction Kit (Shenggong, Shanghai, China), and total RNA was extracted from the treated leaf materials according to the instructions in the RNA Extraction Kit (SUM7806, summer BIOTECH, Beijing, China). The DNA and RNA were tested for quality and concentration with NanoDrop 2000 and Agilent 2100 and then stored at −80 °C. The RNA was used as templates for cDNA synthesis using TransScript^®^ One-Step gDNA Removal and cDNA Synthesis SuperMix (TransGen Biotech, Beijing, China).

qRT-PCR analysis was carried out using the TransStart^®^ Tip Green qPCR SuperMix (TransGen Biotech, Beijing, China) as recommended by the manufacturer. The primers used are listed in Supplementary Table S1. *TaActin* and *AtActin* were selected as internal controls for wheat and Arabidopsis, respectively. All qRT-PCR amplification was carried out for 30 s at 94 °C, followed by 42 cycles of 94 °C for 5 s and 60 °C for 30 s. The relative mRNA level for TaPOR was calculated using the 2^−∆∆t^ method [45]. For each sample, qRT-PCR was performed with three technical replicates from three biological replicate samples.

### 4.6. Analysis of the TaPORs Gene Sequence and Promoter Sequence

The CDS sequences of *TaPORs* were acquired from the EnsemblPlants database and were confirmed using the NCBI database. All cloning primers were designed using the Primer Premier 5.0 software and are presented in Table S3.

To analyze the gene structure of *TaPORs*, KOD-Plus-neo (TOYOBO, Osaka, Japan) was used to amplify the genome sequences (the primers used are listed in Table S1). PCR amplification was carried out for 2 min at 94 °C, followed by 44 cycles of 98 °C for 10 s, 60 °C for 30 s and 68 °C for 90 s with a final extension at 68 °C for 7 min. The PCR products were detected using 1.0% agarose gel and the target bands were purified as previously described. Then, the purified band was cloned into the pGEM-T Easy Vector (Promega, Madison, USA) for sequencing (TSINGKE, Beijing, China). Multiple alignment analysis of the sequences was performed with ClustalX2.1 and GenDoc 2.7 software [46]. The retrieved wheat sequences were used as queries to repeat the step in an iterative manner. Phylogenetic trees were constructed with the program MEGA X (https://www.megasoftware.net/download_form, accessed on 16 June 2021) using the maximum likelihood approach with 1000 bootstrap replicates [47].

The approximately 2000 bp promoter region of the *TaPORs* was obtained from the EnsemblPlants database and design primers. The *TaPORs* promoter sequence was analyzed using the New PLACE database (https://www.dna.affrc. go.jp/PLACE/?action=newplace, accessed on 18 June 2021) and the PlantCARE database (http://bioinformatics.psb.ugent.be/webtools/plantcare/html/, accessed on 18 June 2021).

### 4.7. Construction of Expression Vectors and Ectopic Expression of TaPOR2D in Arabidopsis

The full-length CDS of *TaPOR2D* was amplified from XN1376 using TaPOR-OE-F and TaPOR-OE-R primers (Table S3). The PCR product was cloned into the CaMV 35S promoter-driven expression cassette of pCAMBIA1302 using NcoI and SpeI restriction sites to generate the 35S::TaPOR construct. Arabidopsis Seeds (Col-0) were obtained from our laboratory. The constructs were transformed into Agrobacterium tumefaciens strain GV3101 for Arabidopsis thaliana via the floral dip method [48]. Transgenic lines were grown on 1/2 MS medium supplemented with 50 mg/L hygromycin B. Transcript analysis was performed for the T2 transgenic Arabidopsis lines via qRT-PCR. Based on transcript analysis, representative homozygous T3 progeny were selected for further studies.

### 4.8. Subcellular Localization Assay of TaPOR2D

The full-length CDS of *TaPOR2D* was amplified using gene-specific primers (Table S3). The *TaPOR2D* sequence was cloned into the 35S-EGFP vector with EGFP reporter gene to create a recombinant cassette, wheat 35S::TaPOR-EGFP. Recombinant plasmids were transiently expressed in N. benthamiana leaf cells [49]. After 36 h, tobacco leaf cells were observed on an IX83-FV1200 confocal laser scanning microscope (Olympus, Tokyo, Japan).

### 4.9. The Sodium Bisulfite Treatment and Sequencing

The total DNA amount ranged from 200 to 500 ng per sample. DNA was processed using the Ezup Spin Column Super Plant Genomic DNA Extraction Kit (Sangon, Shanghai, China) and was then stored at −20 °C. Bisulfite processes DNA was processed using the EpiArt DNA Methylation Bisulfite Kit (Vazyme, Nanjing, China) and was then stored at −20 °C. The promoter sequences of *TaPOR2D* for methylation detection are shown in Table S3. Analysis of target sequence CG content and distribution was undertaken using MethPrimer online software (http://www.urogene.org/methprimer2/ accessed on 8 October 2021). The BSP-PCR primers were designed using Methyl Primer software v1.0 and Primer 6 software v6.0, and primer information is as shown in Table S3. The reaction system was 50 μL in total and used the 2 × EpiArt^®^ HS Taq Master Mix (Vazyme, Nanjing, China). The amplification reaction procedure was as follows: predenaturation at 95 °C for 5 min, denaturation at 95 °C for 30 s, annealing at 55 °C for 30 s, and extension at 72 °C for 15 s, with 40 cycles. The PCR product was detected via 2% agarosegel electrophoresis. Sequence information was obtained by cloning the fragments into vector pGEM-T, and 30 clones were sequenced for each fragment.

### 4.10. Statistical Analysis

Statistical significance was evaluated using Student’s *t*-tests. *p*-values < 0.05 and < 0.01 were used as the thresholds for significant and very significant differences, respectively. The Pearson coefficient was calculated via the software SPSS 21.0.

## 5. Conclusions

In conclusion, we found that low temperatures induced albinism in XN1376B. The number of chloroplasts decreased and fence tissue and sponge tissue slowly dissolved when XN1376B entered its albino stage. We identified *TaPOR2D*, which was deemed to be related to the phenomenon of albinism based on its expression in different colored leaves. Our results showed that promoter methylation is associated with the expression of *TaPOR2D*, affecting the level of chlorophyll accumulation in albino wheat and the development of plant chloroplasts, and probably causes the albino phenotype of XN1376B at low temperatures. The results provide a theoretical basis for in-depth analysis of the regulation of development of plant chloroplasts and color variation in wheat XN1376B leaves.

## Figures and Tables

**Table 1:** Transformation of leaf color in the field.

Development	Temperature (°C)	Leaf Color of XN1376	Leaf Color of XN1376B
before two-leaf stage	10–15	green	green
three-leaf stage	5–10	green	white striped
four-leaf stage	0–5	green	white
return green stage	10–15	green	return-green

## Data Availability

The data underlying this article are available in the article and online supplementary material.

## Supplementary Materials

The following supporting information can be downloaded at https://www.mdpi.com/article/10.3390/ijms241914697/s1.
