# Supplementary material for: Low-Temperature-Mediated Promoter Methylation Relates to the Expression of TaPOR2D, Affecting the Level of Chlorophyll Accumulation in Albino Wheat (Triticum aestivum L.)
Source: Int J Mol Sci. 2023 Sep 28;24(19):14697. doi: 10.3390/ijms241914697 (PMC10573025; doi:10.3390/ijms241914697)
Supplement: Supplementary file 1 [file ijms-24-14697-s001.zip › Captions.pdf]

Captions

1. Table 1. Transformation of leaf color in the field.

2.Table S1. Features of the wheat (*Triticum aestivum* L.) POR genes.

3.Table S2. Correlation analysis of *TaPOR2D* gene expression and promoter methylation rate under different leaf colors of XN1376B.

4.Table S3. Primers in this study.

5. Figure 1. Phenotype and chlorophyll content of XN1376 and XN1376B. A-B: XN1376B change characteristics.1. two-leaf stage; 2. three-leaf stage; 3. four-leaf stage. C: overwintering stage of XN1376 and XN1376B; D: chlorophyll content of XN1376 and XN1376B.

6.Figure 2. ultrastructural of mesophyll cells in XN1376B, A, D, G: white leaf of XN1376B; C, F, I: green leaf of XN1376; E: Epidermis, M: Mesophyll, C: Chloroplast, Pt: Palisade tissue, St: Spongy tissue. Scale bar: 200 μm in (A-C), 100 μm in (D-I).

7.Figure 3. Phylogenetic, conserved motifs and gene structure of POR families from wheat, sorghum, barley, rice, maize and Arabidopsis.

8.Figure 4. Expression profiles of 6 *TaPOR* genes in green and white leaves. 1376G: green leaf, 1376W: white leaf, 1376RG: return-green leaf.

9.Figure 5. Main cis-elements in TaPOR gene promoters. (A) Light responsive, abiotic stress-related, hormone-related, development-related and transcription-related elements were identified in the TaPOR gene promoter regions. (B) Location of 16 type light response elements and cryogenic elements in the *TaPOR*s promoter region.

10.Figure 6. Subcellular localization of TaPOR2D fused with EGFP in the epidermal cells of *N. benthamiana*. The selected TaPOR genes were cloned from XN1376 and used to construct CaMV35S::TaPORs–EGFP vectors in which EGFP was fused at the C-terminus. Bar =40 μm.

11.Figure 7. Phenotypic characterization of transgenic and wild-type (WT) Arabidopsis plants. (A) Representative images show WT and transgenic lines, after one week of growth, two weeks of growth, three weeks of growth, and four weeks of growth. (A) qRT-PCR identification of transgenic TaTDRL overexpression

Arabidopsis plants. (C) Total content of chlorophyll in WT, TaPOR2D-overexpressing (OE-1 and OE-2). At Actin was used as an internal control. Data represent means ± SD (n = 3). Each column represents the mean ± standard error based on three biological repeats.

12.Figure 8. Methylation profiles in the promoter regions of *TaPOR2D*. A: Green leaves; B: White leaves.

13.Figure 9. Expression profiles of 3 methyltransferases in XN1376B. Quantitative real time polymerase chain reaction (RT-qPCR) was used to detect the expression levels of the TaMET1, TaCMT, TaDRM genes. TaActin was used as an internal control. Each value represents the mean of three biological replicates ± SE. 1376G: green leaf, 1376W: white leaf, 1376RG: return-green leaf.

14.Figure S1. Multi-sequence comparison diagram of wheat POR proteins.
red box, adh_short domain.

15.Figure S2. romoters of 6 *TaPOR* genes amplification electrophoresis.

16.Figure S3. Prediction of CpG islands in the promoter regions of *TaPOR2D* genes.
