# Supplementary material for: Low-Temperature-Mediated Promoter Methylation Relates to the Expression of TaPOR2D, Affecting the Level of Chlorophyll Accumulation in Albino Wheat (Triticum aestivum L.)
Source: Int J Mol Sci. 2023 Sep 28;24(19):14697. doi: 10.3390/ijms241914697 (PMC10573025; doi:10.3390/ijms241914697)
Supplement: Supplementary file 1 [file ijms-24-14697-s001.zip › supplementary materials.pdf]

Table S1. Features of the wheat (*Triticum aestivum* L.) POR genes

NAME	Locus ID	Chromosome location	Orientation	Peptide length(a.a.)	Intron	pI	MW(kDa)	Subcellular localization
TaPOR1A	TraesCS1A02G171000	1A:305607006-305608049	Forward	417	3	9.25	42.23	Chloroplast
TaPOR1B	TraesCS1B02G186300	1B:333322305-333323368	Reverse	395	3	9.25	42.22	Chloroplast
TaPOR1D	TraesCS1D02G168700	1D:240989549-240990597	Forward	417	3	9.15	44.72	Chloroplast
TaPOR2A	TraesCS2A02G590600	2A:778723494-778724270	Reverse	388	1	9.37	41.24	Chloroplast
TaPOR2B	TraesCS2B02G593000	2B:777828926-777829703	Forward	388	2	9.42	41.15	Chloroplast
TaPOR2D	TraesCS2D02G563600	2D:634796733-634797988	Forward	388	1	9.42	41.24	Chloroplast

Table S2. Correlation analysis of *TaPOR2D* gene expression and promoter methylation rate under different leaf colors of XN1376B

	Gene expression	Methylation rate
XN1376G	1	
XN1376W	-1	1

Table S3. Primers in this study

Gene	Forward Primer（5’ - 3’）	Reverse Primer（5’ - 3’）
TaPOR1A	GATGGCCTCAAATTCGACAC	CTCTGCGTAGGTTCGTGAC
TaPOR1B	CACCGCACTATCTGATACACTC	GCCTGAAGAGCCATCTGAG
TaPOR1D	CGCACTATCTCATACACTCGTC	CCATCGGCGAGACAAACG
TaPOR2A	GAGCTCACCGTCATCCATG	GTCCTTCACTGCCACCG
TaPOR2B	GTACTGGAGCTGGAACAAGG	TCCCGCGTAATTGAGTTTCTG
TaPOR2D	GAGCTCACCGTCATCCATG	ACGCCTTCTTCGCCTTG
TaActin	ACCTTCAGTTGCCCAGCAAT	CAGAGTCGAGCACAATACCAGTTG
AtActin	GGTAACATTGTGCTCAGTGGTGG	AACGACCTTAATCTTCATGCTGC
BSP1	GGGTTTATATATTTTTATTTATGTAGGTTA	CTCCACAACCAATCACAAAAC
BSP2	TGTTATGGAGGTTGTTTTATTTGATAAG	CCTACATAAATAAAAATATATAAACCC
BSP3	ATTGGTTGTGGAGGTG	ACTATCGCTAACAAACTC
TaMET1	GGCACAATCAGTGGAAGGG	GCCCGAAAACTGGTAGCTG
TaCMT	GGTCGGTTGTGGTGGGATG	CATACCCCACATCCACCTCAC
TaDRM	TGAGTGCTCGCCCTCCAAG	TTAGACGATTTGGCGGCTTC
TaPOR2D-EGFP	CGAGCTCAAGCTTCGAAATGGGAGGAGGAGATTATCACC	CGACTGCAGAATTCGAAATCCATGGCGAGGTACTGCAG

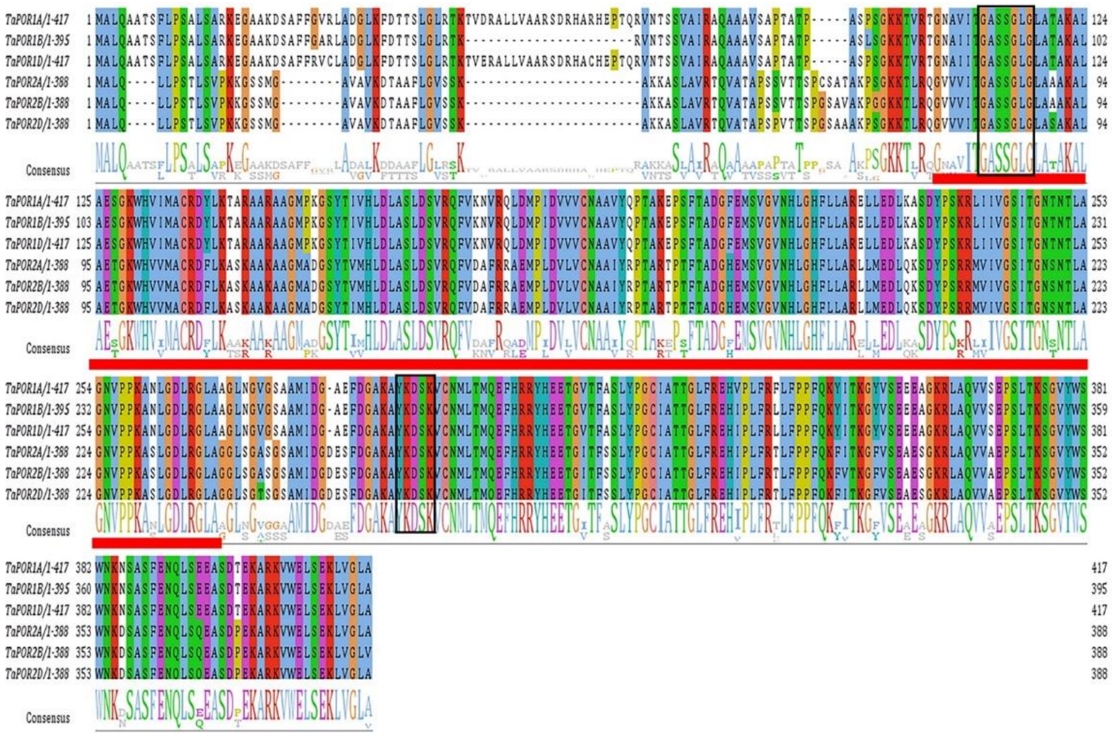

Figure S1. Multi-sequence comparison diagram of wheat POR proteins.
red box, adh_short domain

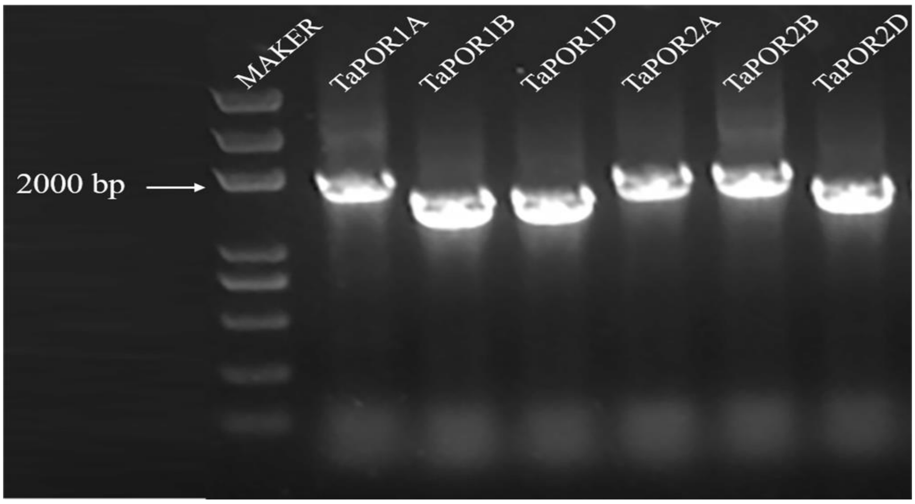

Figure S2. romoters of 6 *TaPOR* genes amplification electrophoresis

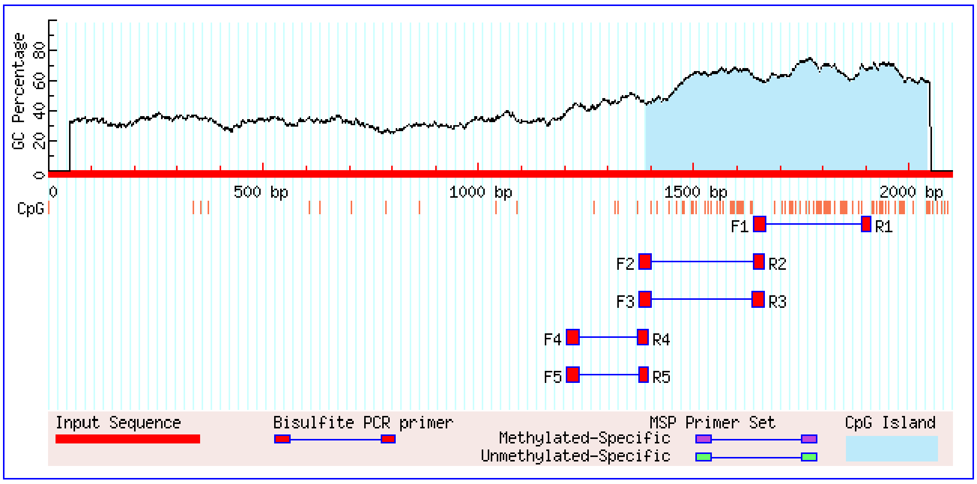

Figure S3. Prediction of CpG islands in the promoter regions of *TaPOR2D* genes.
