# Supplementary figures and images for: Low-Temperature-Mediated Promoter Methylation Relates to the Expression of TaPOR2D, Affecting the Level of Chlorophyll Accumulation in Albino Wheat (Triticum aestivum L.)
Source: Int J Mol Sci. 2023 Sep 28;24(19):14697. doi: 10.3390/ijms241914697 (PMC10573025; doi:10.3390/ijms241914697)

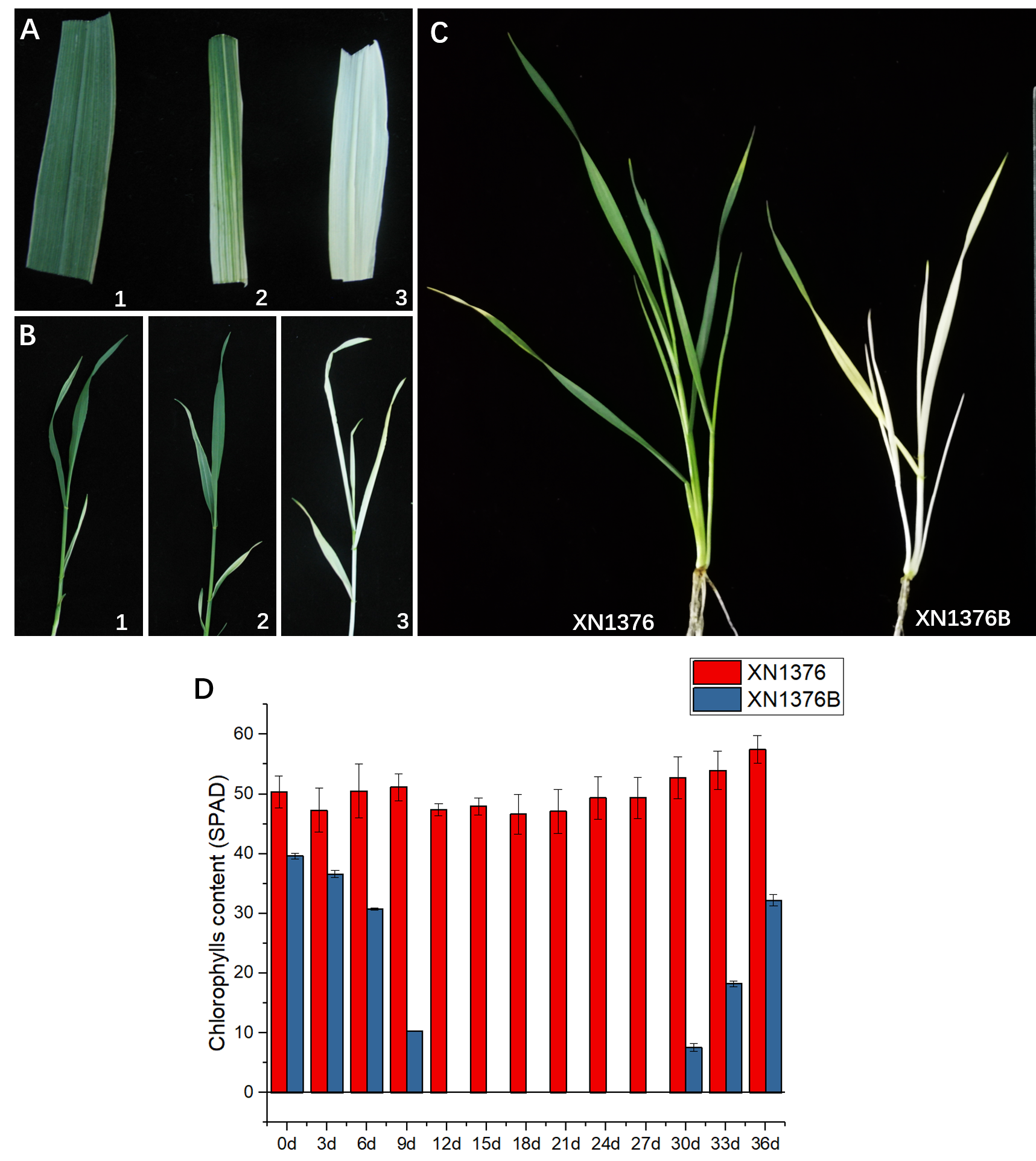

Supplement: Supplementary file 1 [file ijms-24-14697-s001.zip › Figure1.tif]

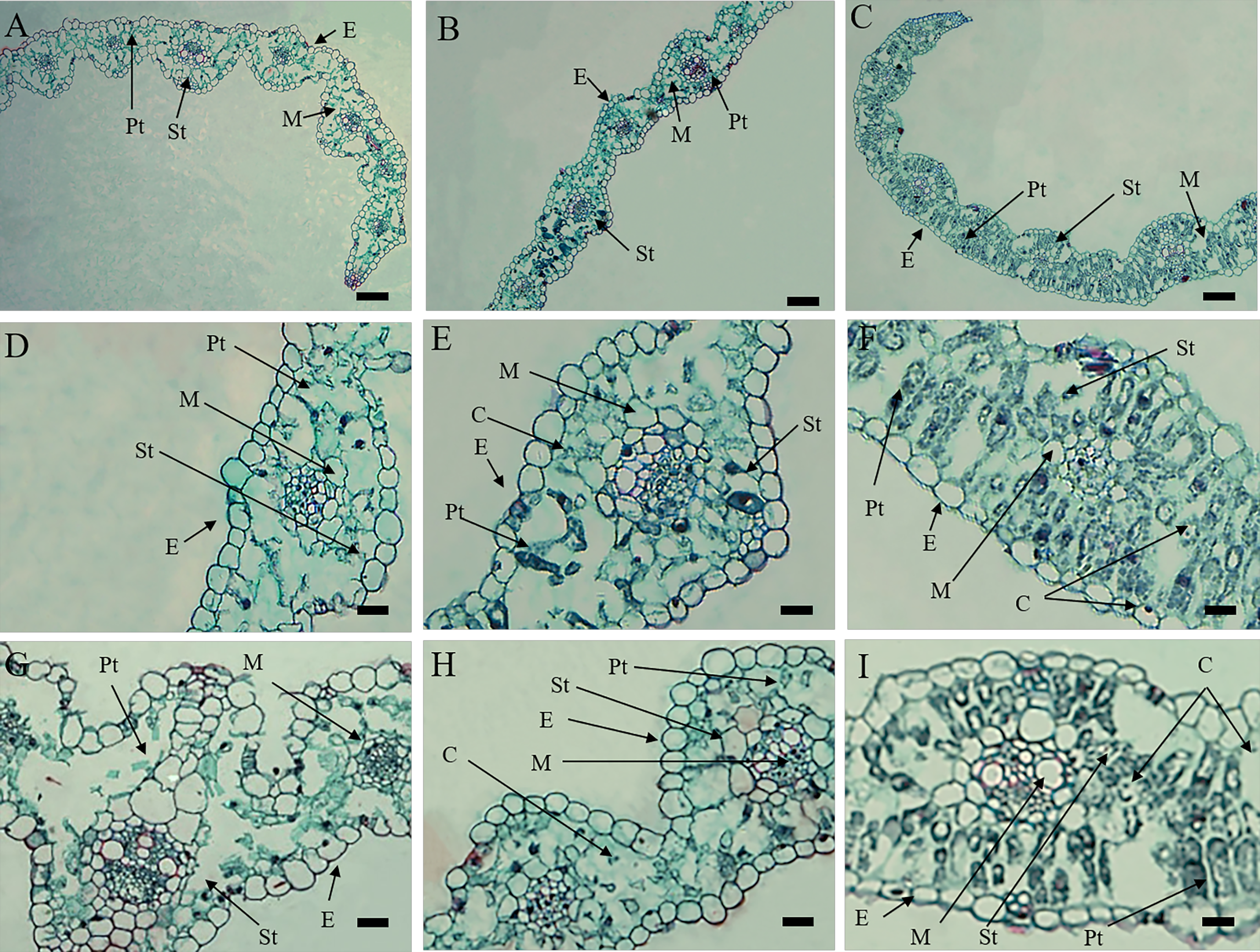

Supplement: Supplementary file 1 [file ijms-24-14697-s001.zip › Figure2.tif]

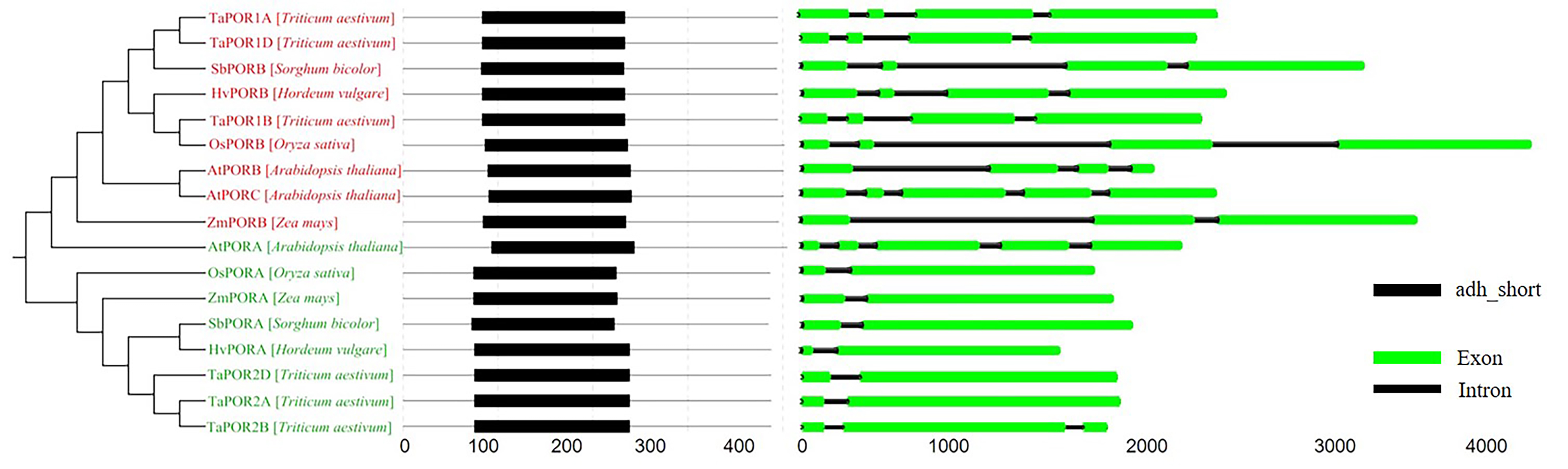

Supplement: Supplementary file 1 [file ijms-24-14697-s001.zip › Figure3.jpg]

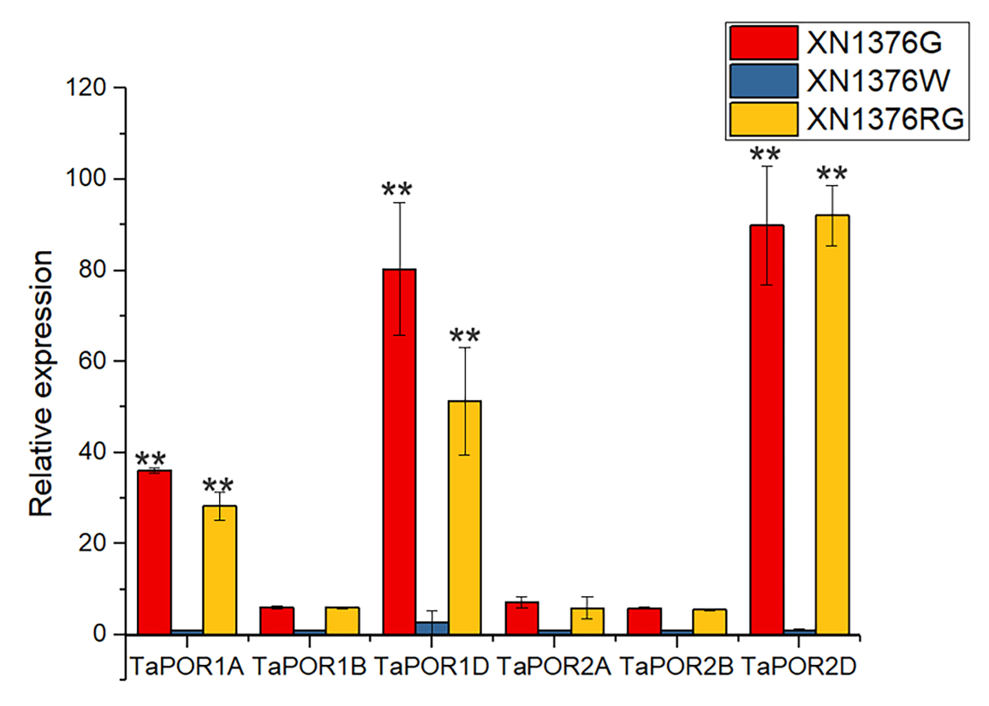

Supplement: Supplementary file 1 [file ijms-24-14697-s001.zip › Figure4.tif]

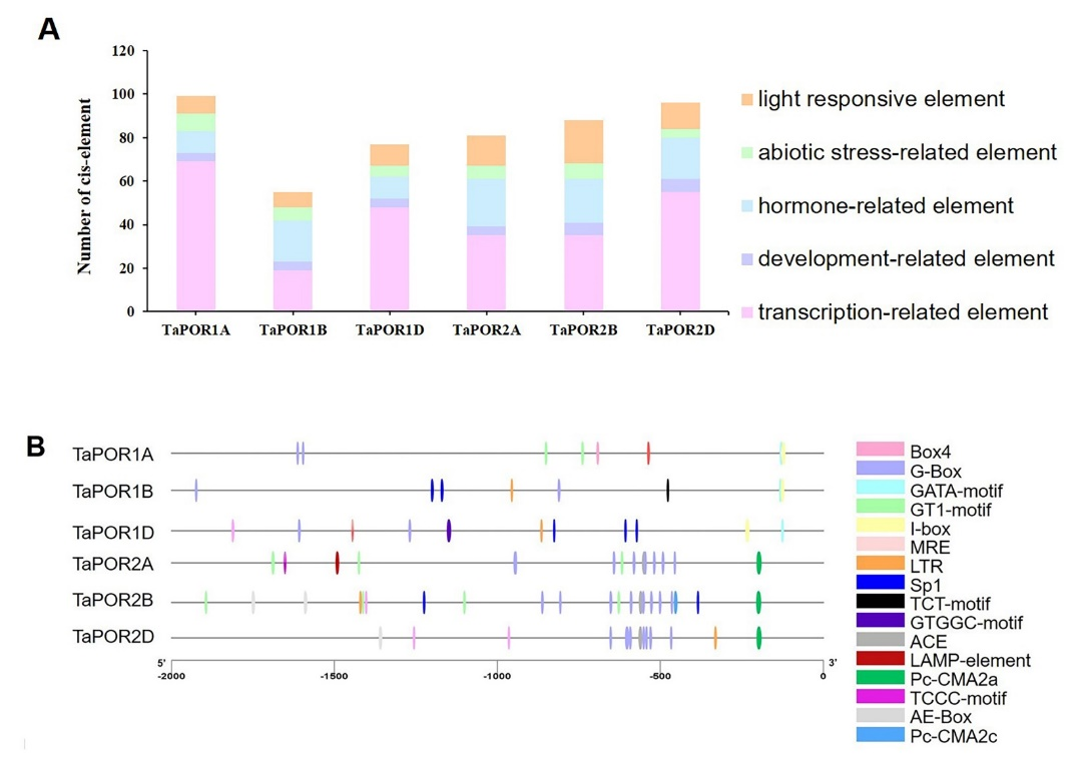

Supplement: Supplementary file 1 [file ijms-24-14697-s001.zip › Figure5.tif]

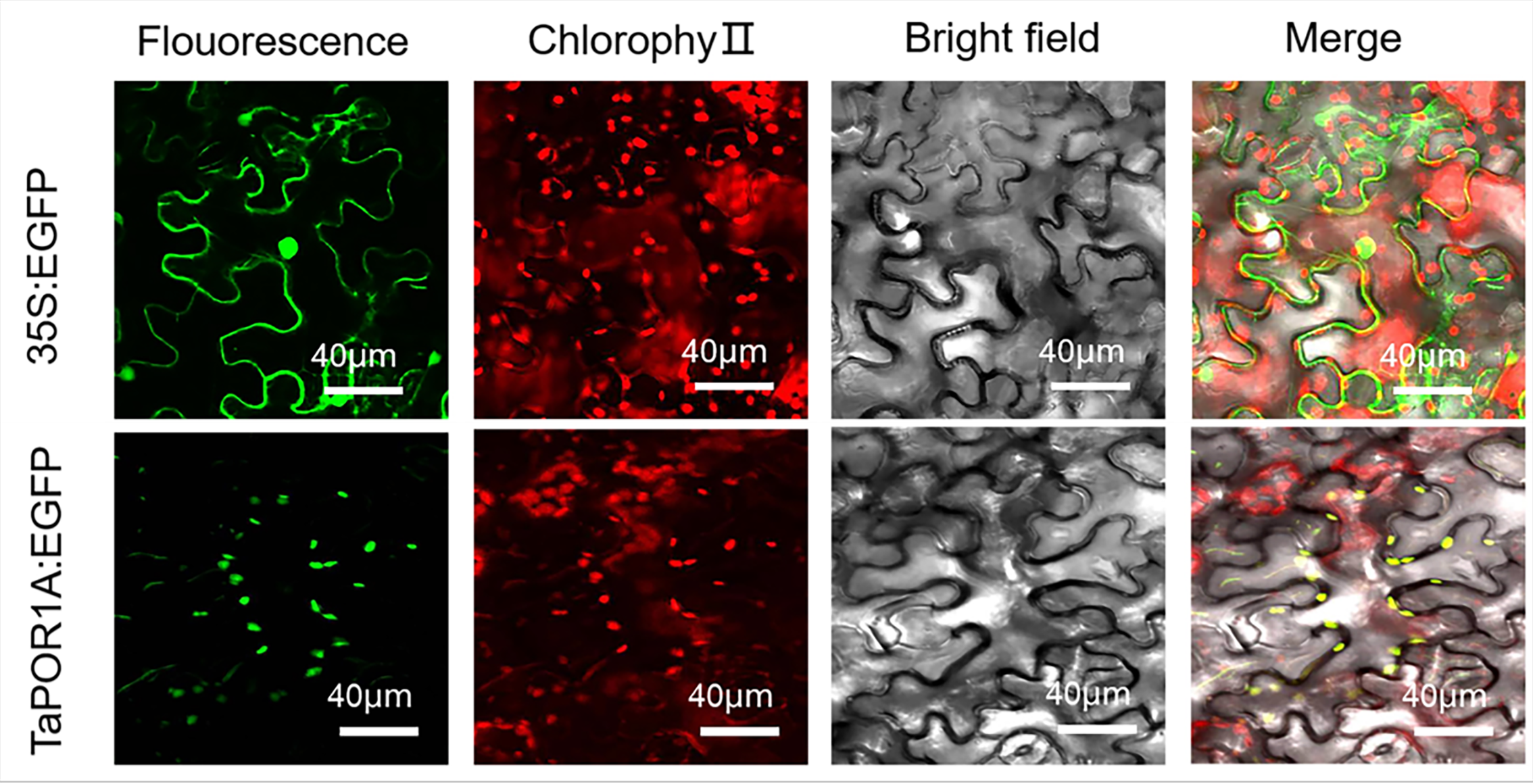

Supplement: Supplementary file 1 [file ijms-24-14697-s001.zip › Figure6.tif]

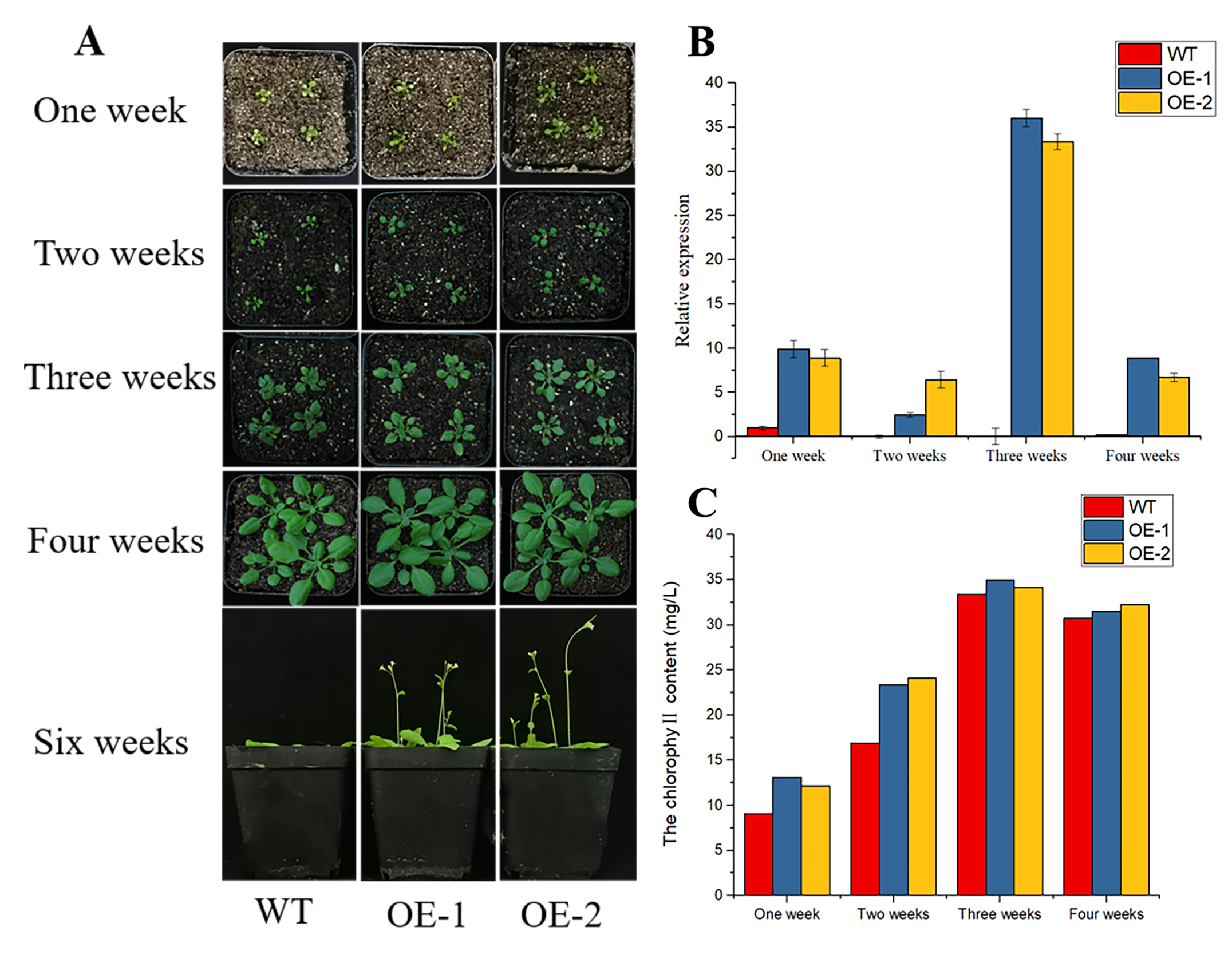

Supplement: Supplementary file 1 [file ijms-24-14697-s001.zip › Figure7.tif]

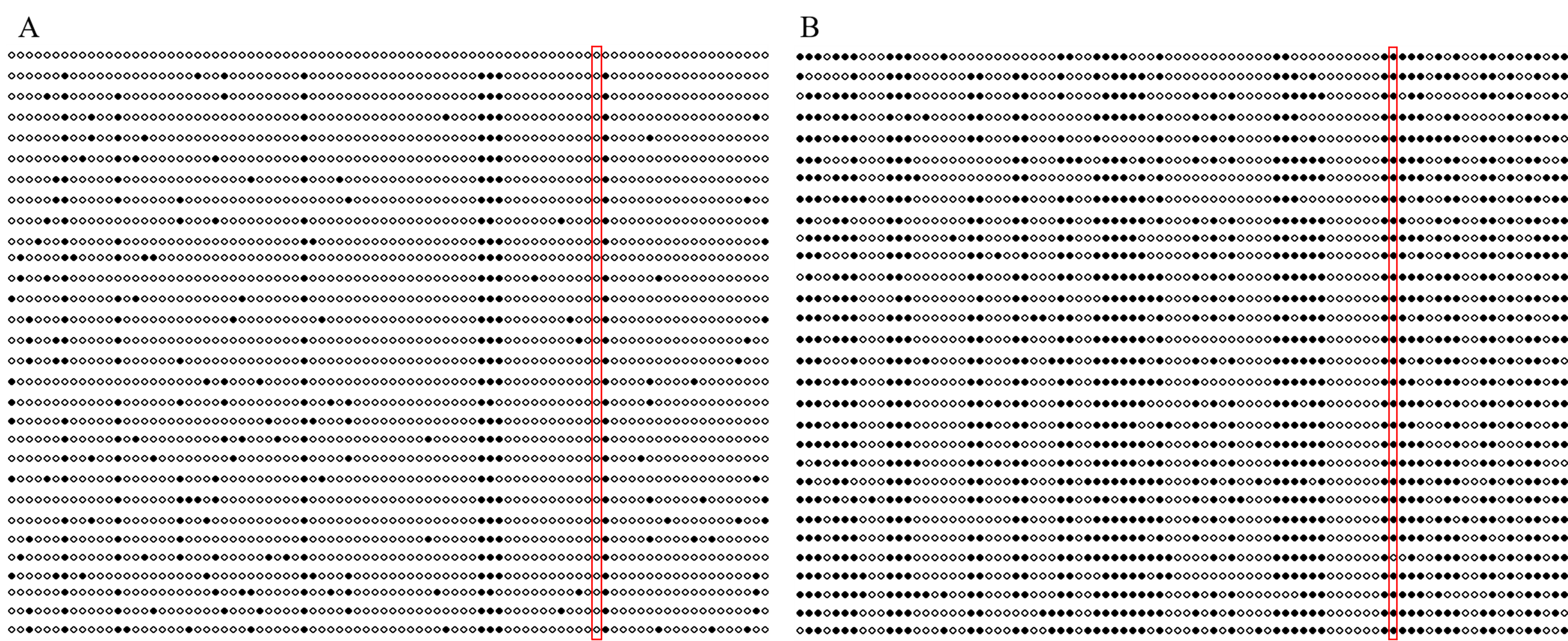

Supplement: Supplementary file 1 [file ijms-24-14697-s001.zip › Figure8.tif]

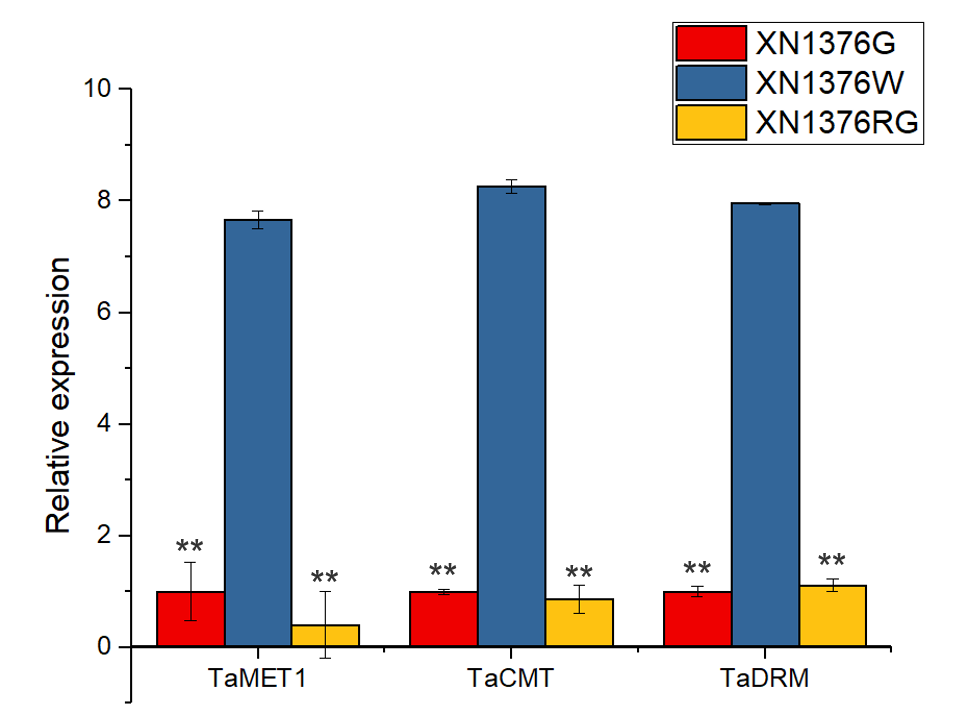

Supplement: Supplementary file 1 [file ijms-24-14697-s001.zip › Figure9.tif]

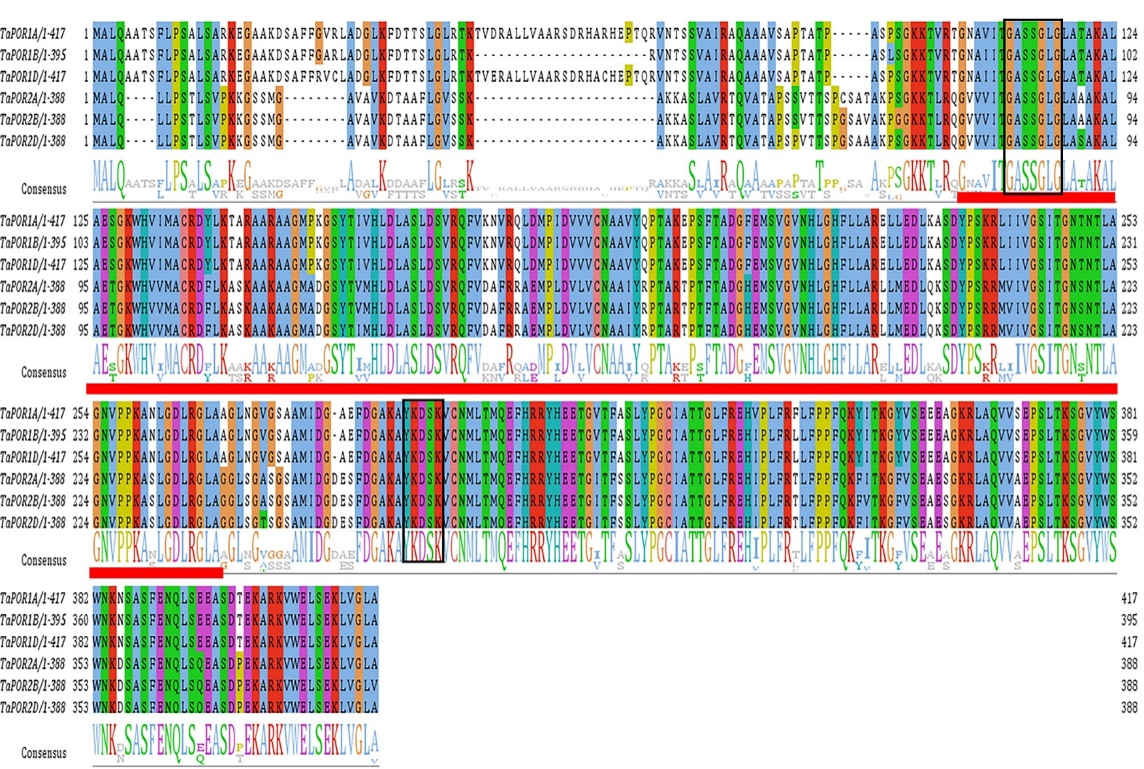

Supplement: Supplementary file 1 [file ijms-24-14697-s001.zip › FigureS1.tif]

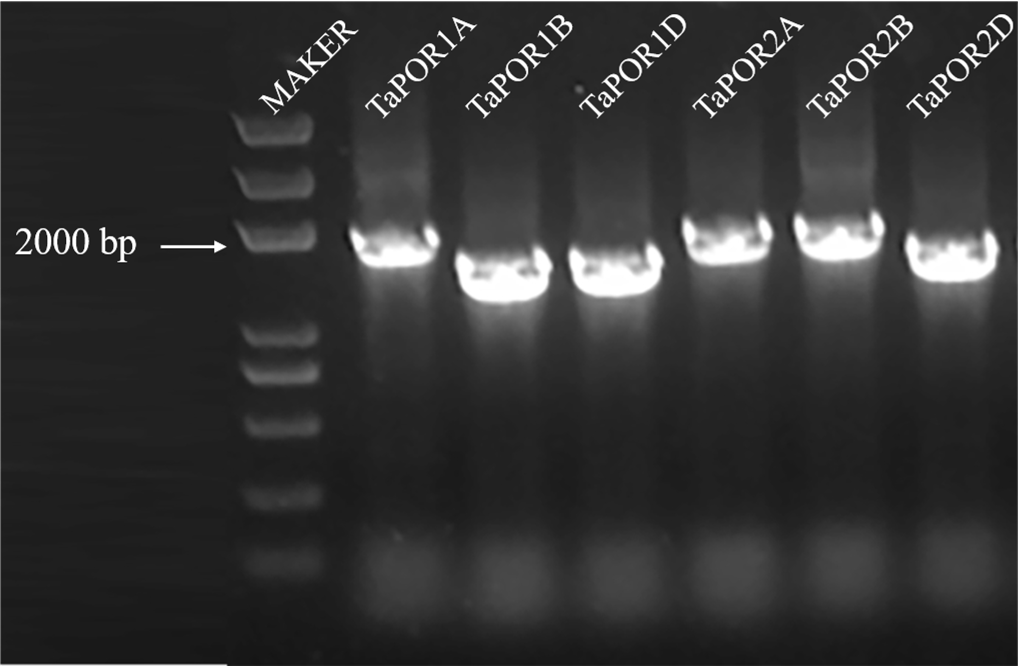

Supplement: Supplementary file 1 [file ijms-24-14697-s001.zip › FigureS2.tif]

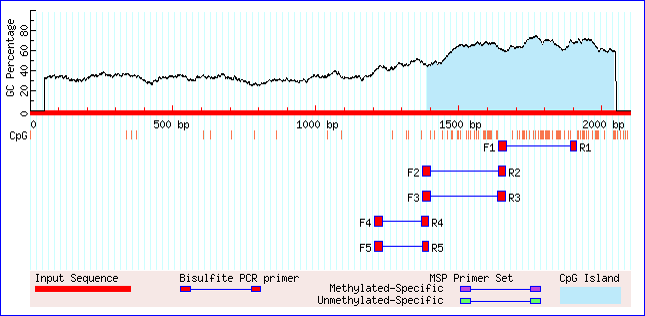

Supplement: Supplementary file 1 [file ijms-24-14697-s001.zip › FigureS3.tif]
